# Congenital vertical talus in Cri du Chat Syndrome: a case report

**DOI:** 10.1186/1756-0500-6-270

**Published:** 2013-07-13

**Authors:** Amani Khader, James S Huntley

**Affiliations:** 1 University of Glasgow, University Avenue, G12 8QQ, Glasgow, United Kingdom; 2 Consultant Orthopaedic Surgeon, Royal Hospital for Sick Children, G3 8SJ, Yorkhill, Glasgow, United Kingdom

**Keywords:** Congenital vertical talus, Cri du chat syndrome, Flat foot

## Abstract

**Background:**

Congenital vertical talus is a rare deformity of the foot which can cause substantial pain and disability. Its incidence is approximately 1 in 100,000 live births. It has an association with other neuromuscular abnormalities and identified genetic syndromes in 50% of cases [1-5]. This report presents a case of congenital vertical talus in an infant with Cri du Chat Syndrome (CdCS) which - to our knowledge - has not been previously reported.

**Case presentation:**

A 2 week-old Caucasian, male infant was referred for congenital feet abnormalities and a “clicky” hip at the post-natal baby check. The diagnosis was vertical talus of the right foot and oblique talus of the left foot. Treatment involved serial plaster casts in the “reverse-Ponseti” position until surgery 16 weeks later. The correction was maintained and the feet remain in good position at follow-up. General concern over the infant’s development, failing to reach appropriate milestones, prompted paediatric referral. Genetic analysis was finally carried out, giving a diagnosis of Cri du Chat syndrome at two and a half years of age.

**Conclusion:**

In light of other reports of chromosomal anomalies causing congenital vertical talus, the learning point from this case is to investigate early for possible aetiologies, not only spinal/neuromuscular, but also those of a genetic basis.

## Background

Congenital vertical talus is a rare deformity of the foot, characterised by a rigid flatfoot/rocker bottom foot deformity [1-3]. It results from a fixed dislocation of the talonavicular joint and an equinus hindfoot. If untreated it can cause substantial pain and disability. Its incidence is approximately 1 in 100,000 live births, with no sex predilection, and it has association with neuromuscular abnormalities and identified genetic syndromes in 50% of cases [1-5]. This report presents a case of congenital vertical talus in an infant with Cri du Chat Syndrome (CdCS), which - so far as we are aware - has not been previously reported in literature.

## Case presentation

A 2 week-old Caucasian, male infant was referred for congenital feet abnormalities and a “clicky” hip at the post-natal baby check. He had been delivered by elective caesarean section at 39 weeks, with no complications. He had appeared small for dates at the 12 and 20- week routine antenatal ultrasounds and had a birth weight of 5 lb 9 oz. He was the 2nd son (older half brother aged 4 years), with no family history of note.

On examination, the infant had “bean-shaped” rocker-bottom feet (left foot more flexible than right) with a right mid-foot break and excessive posterior prominence. He had bilateral up-going plantar responses. The hips were normal on both clinical and ultrasonographic examination, and the spine was straight with no palpable anomalies, pigmentations or hairy patches, and only the presence of a very small sacral dimple right side to the midline. Spinal ultrasound was normal but yielded an incidental finding of abnormal kidneys, differentially a horseshoe kidney or cross-fused renal ectopia, and it was planned for these to be evaluated further with an interval Dimercaptosuccinic acid (DMSA) scan.

The diagnosis was vertical talus of the right foot and oblique talus of the left foot. It was planned to carry out bilateral reverse Ponseti casting followed by limited surgery (open talonavicular reduction and wiring). A neurology opinion was obtained because of the known association between neuromuscular abnormalities and vertical tali. It returned no abnormal features.

Treatment involved plaster casts in the reverse-Ponseti position (plantar-flexion and medial deviation), with weekly recasting to reduce the talonavicular joints until surgery 16 weeks later. Casting was thought to have reduced the left side after 2 weeks, whilst the right side remained dislocated.

### Surgical correction

**Figure 1:**
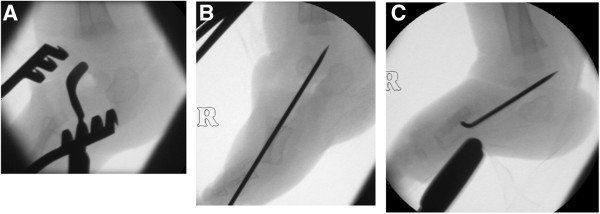
**X-ray series showing surgical correction and stabilisation - intraoperative lateral image intensifier views of right foot. (A)** A dorsomedial incision has been used to approach the talonavicular joint. Note the obliquity of the bean-shaped ossification centre of the talus. **(B)** The talonavicular joint has been reduced. Note the new axis of the talar ossification centre. **(C)** The reduction is maintained as the ankle is dorsiflexed after tendo Achilles lengthening.

Surgical correction involved bilateral open talonavicular reduction, stabilisation with threaded Kirschner-wires and Achilles tenotomies (Figure 1). The right foot was more complex, requiring more extensive talonavicular release to allow reduction. Finally, long leg casts were applied. Post-operatively, casts were changed at 18 days, and the left wire removed at 5 weeks, and the right removed at 8 weeks.

### Follow-up

The correction was maintained and the feet remained in good positions at each follow-up (now at 3 years), and orthotic casts (boots and bar) were made to maintain position. General concern over the infant’s development, failing to reach appropriate milestones, namely sitting up, sitting up unsupported, and cruising, prompted paediatric referral. At first instance, it was suggested that this might simply be a direct consequence of his orthopaedic and surgical interference delaying his gross motor development. However, increasingly, worry for other areas of development (including both cognitive and fine motor) grew, as he failed to reach milestones. Genetic analysis was finally carried out, giving a diagnosis of Cri du Chat syndrome at two and a half years of age.

## Conclusion

Congential vertical talus is a rare, potentially disabling deformity of the foot, characterised by a rigid flatfoot. Primarily, it has been linked to disorders such as distal arthrogryposis and myelomeningocele, and an array of chromosomal abnormalities, single gene defects and known genetic conditions [5] (Table 1).

**Table 1:** **Congenital aetiologies**^**1,5**^**: reported cases**

**Neuromuscular**	
•	Central nervous system: cerebral palsy, myelomeningocele, caudal regression syndrome, hydrocephalus
•	Muscular: arthrogryposis, multiple pterygium syndrome, neurofibromatosis
**Genetic**	
•	Chromosomal: trisomy 13, trisomy 15, trisomy 18, trisomy 21, 12q duplication, 16p13.3 duplication
•	Single gene defects: HOXD10, CDMP1, GDF5
•	Known syndromes: neurofibromatosis, Down Syndrome, Prune-Belly syndrome, Rasmussen syndrome, Costello syndrome, De Barsy syndrome, split hand and split foot

Despite the growing number of clinical syndromes linked to the occurrence of congenital vertical talus, there appears to be no record of literature illustrating its presence in Cri du Chat Syndrome (CdCS). CdCS, named because of the characteristic high pitched “cat-like” crying present in the affected infant’s first year of life, is a clinical syndrome arising from a partial or total genetic deletion on the short arm of chromosome 5 (5p15) [6-9]. It has an incidence of 1/15,000-1/50,000 live births [6,8,9], with a slightly higher female occurrence and is recognised by the landmark features of a high pitched cry, dysmorphism, microcephaly and mental retardation [6-10]. The majority of cases (approximately 80%) arise from a *de novo* deletion, 10% occur due to a parental translocation, and fewer than 10% result from rare aberrations, and the location and size of the pathogenic deletion ranges across the span of the whole chromosome short arm [6].

Interestingly, variations in the genotypic mutation concordantly give rise to variability in the phenotype, and different sites have been mapped to particular phenotypes, with literature identifying this mutagenic link with various anomalies and resultant clinical severity [6-8]. It has already been recognised that a deletion in the 5p15.3 region results in the characteristic cry, whereas deletions in the 5p15.2 region give rise to the other features of dysmorphism, microcephaly and mental handicap [7,8]. With significance to this case, few orthopaedic abnormalities have been described, asides from scoliosis that has been found in numerous cases [11].

The possibility that other orthopaedic anomalies may present in CdCS is plausible, and recognising them may prompt clinicians to an earlier diagnosis of CdCS. For this infant, a clue may have been the early findings of an abnormal kidney, a feature that has been linked to not only CdCS, but also various other genetic conditions and could have prompted quicker genetic investigation.

In light of other reports of chromosomal anomalies causing congenital vertical talus, the learning point from this case is to investigate early for possible aetiologies, not only spinal/neuromuscular, but also those of a genetic basis, and not rely on speciality practitioners for this.

### Consent

Written informed consent was obtained from the infant’s parents for publication of this case report and its accompanying images.

## Abbreviations

CdCS: Cri du chat syndrome.

## Competing interests

The authors declare that they have no competing interests.

## Authors’ contributions

JSH looked after the patient and conceived of the idea for the report after a limited literature review. AK reviewed the literature and wrote the first draft. JSH and AK together revised and rewrote the manuscript. JSH is the guarantor. All authors read and approved the final manuscript.
